# Risks associated with endotoxins in feed additives produced by fermentation

**DOI:** 10.1186/s12940-016-0087-2

**Published:** 2016-01-15

**Authors:** R. John Wallace, Jürgen Gropp, Noël Dierick, Lucio G. Costa, Giovanna Martelli, Paul G. Brantom, Vasileios Bampidis, Derek W. Renshaw, Lubomir Leng

**Affiliations:** Rowett Institute of Nutrition and Health, University of Aberdeen, Bucksburn, Aberdeen, AB21 9SB UK; Universität Leipzig, Leipzig, Germany; Department of Animal Production, Ghent University, Ghent, Belgium; Department of Neuroscience, University of Parma, Parma, Italy; Department of Environmental and Occupational Health Sciences, University of Washington, Seattle, WA USA; Department of Veterinary Medical Sciences, University of Bologna, Bologna, Italy; Brantom Risk Assessment Ltd, Crawley, UK; Division of Animal Production, Department of Agricultural Technology, School of Agricultural Technology, Food Technology and Nutrition, Alexander Technological Educational Institute (ATEITHE), 57400 Thessaloniki, Greece; Independent consultant on toxicology, Ladywell, London, UK; Institute of Animal Physiology, Slovak Academy of Sciences, Kosice, Slovakia

**Keywords:** Endotoxin, Feed additives, Lipopolysaccharide, Worker exposure

## Abstract

Increasingly, feed additives for livestock, such as amino acids and vitamins, are being produced by Gram-negative bacteria, particularly *Escherichia coli*. The potential therefore exists for animals, consumers and workers to be exposed to possibly harmful amounts of endotoxin from these products. The aim of this review was to assess the extent of the risk from endotoxins in feed additives and to calculate how such risk can be assessed from the properties of the additive. Livestock are frequently exposed to a relatively high content of endotoxin in the diet: no additional hazard to livestock would be anticipated if the endotoxin concentration of the feed additive falls in the same range as feedstuffs. Consumer exposure will be unaffected by the consumption of food derived from animals receiving endotoxin-containing feed, because the small concentrations of endotoxin absorbed do not accumulate in edible tissues. In contrast, workers processing a dusty additive may be exposed to hazardous amounts of endotoxin even if the endotoxin concentration of the product is low. A calculation method is proposed to compare the potential risk to the worker, based on the dusting potential, the endotoxin concentration and technical guidance of the European Food Safety Authority, with national exposure limits.

## Background 

Gram-negative bacteria are characterised by having an outer membrane in which a structural component is lipopolysaccharide (LPS). All Gram-negative bacteria contain LPS. LPS varies in chemical structure, particularly in polysaccharide composition, across Gram-negative species and even among strains of the same species [1–3]. LPS has endotoxin activity, whose potency varies enormously among species and their different LPS structures, particularly lipid A [1–3]. *Escherichia coli* produces a LPS with exceptionally high endotoxin activity [1, 2].

Feed additives to be used in the European Union must be assessed by the European Food Safety Authority (EFSA; http://www.efsa.europa.eu/), before they are authorised. Among the criteria assessed are safety for the target animal, safety for the consumer and safety for the user (workers). Most additives produced by fermentation are derived from fungi and Gram-positive bacteria, none of which contain LPS, although some fungi may contain endotoxin-like activity. However, an increasing number of amino acid products is appearing that derives from fermentation with Gram-negative bacteria, particularly *E. coli*, because genetic systems are well characterised in this bacterium. *E. coli* K-12 is considered safe in most respects, such as the absence of antibiotic production, enterotoxins and virulence factors [4, 5]. However, this long established strain, often considered a ‘laboratory cripple’ [6], still contains LPS, because it is an essential structural component of the outer membrane. LPS of *E. coli* K-12 is less potent than most other strains, nevertheless it retains at least one-quarter of the endotoxin activity of wild-type strains [7–9]. Thus, additives produced by fermentation using *E. coli* K-12 have the potential to be hazardous if LPS passes into the additive. The aim of this paper was to determine the extent of the risk to workers handling the additive, to the consumer and to the target animals from endotoxins in feed additives and to calculate how such risk can be assessed from the properties of the feed additive.

## Toxicity of LPS

### Systemic toxicity

Extremely small amounts of endotoxin (1–4 ng/kg body weight (bw)) reaching plasma can cause severe systemic effects in man [10]. When LPS is administered intravenously, it causes a dose-related increase in serum C-reactive protein, TNF-α, IL-1β,and IL-6, which further causes severe fever, diarrhoea, vomiting, and hypotension [10]. Endotoxin plays a central role in the pathogenesis of septic shock in man [11]. The application of intravenous endotoxin in humans induces a variety of acute inflammatory responses similar to the early stages of septic shock [11]. Changes occur in systemic haemodynamics, ventricular function, pulmonary gas exchange and permeability. A wide variety of inflammatory mediators are released which appear to contribute to these responses. These include the release of proinflammatory cytokines, including TNF-α, IL-1β, IL-6, and IL-8, activation of the fibrinolytic system, kallikrein-kinin generation and phospholipase A2 release [11]. LPS is known to be a very potent antigen and, as a result, stimulates an intense host inflammatory response in man [2, 12] and animals [13], including fish [14]. In cattle, intravenous administration of 0.4 μg of *E. coli* endotoxin per kg bw resulted in many of the symptoms of subacute ruminal acidosis (SARA), including inflammation, ruminal pH declining and changes in the microbial community [15]. Thus, if endotoxin enters into the circulation, severe pathological responses may occur.

### Toxicity by oral ingestion

In their review, the Dutch Committee on Occupational Standards concluded that evidence that oral ingestion of endotoxins causes harm was weak in normal, healthy people [16]. Indeed, oral administration of LPS may even be therapeutic in allergic and lifestyle-related diseases [17]. In contrast, systemic exposure to endotoxins escaping from the gut lumen can be associated with severe inflammatory disease in man [2, 18] and animals [19–23]. Healthy individuals carry a large intestinal load of LPS with no harm, and it is generally accepted that in order for the pathogenesis to occur, the barrier function of the gut must firstly be compromised, as in inflammatory bowel disease in man [2, 24], stress in pigs [25], nematode infection in mice [26] and sub-acute ruminal acidosis (SARA) in cattle [22]. High endotoxin concentrations (>10^5^ IU/ml; [20]) in the rumen are associated with, but insufficient to cause, increases in acute phase proteins serum amyloid-A, haptoglobin, and LPS-binding protein in peripheral circulation usually associated with SARA [21, 22]. The low pH that accompanies SARA increases the permeability of the rumen epithelium [27, 28], and may be necessary for systemic toxicity to take place.

### Effects of endotoxin inhalation

There is abundant evidence in the literature that workers exposed to high endotoxin levels by inhalation suffer impaired lung function. The Dutch expert Committee on Occupational Safety [29] summarised the evidence as follows. “The inhalation of endotoxins may cause the following acute symptoms: dry cough, dyspnoea accompanied by diminished lung function, fever and general malaise. After several hours, the following symptoms may develop: bronchoconstriction, headache and aching joints. The acute effects have been observed in the context of research with volunteers and reported in the outcomes of epidemiological research amongst occupationally exposed people. It has been demonstrated that, in asthma sufferers and people with inflammations of the nasal mucosa, exposure to LPS can lead to bronchial obstruction, accompanied by increased reactivity. Epidemiological research has produced evidence to suggest that prolonged exposure to endotoxins may lead to chronic bronchitis and diminished lung function”.

Workers in sewage plants, poultry sheds, sawmills and materials recycling facilities [29, 30] are particularly exposed to high levels of respirable endotoxins, which leads to chronic bronchitis and diminished lung function [29]. Thorn [31] concluded that inhalation of 30–40 μg LPS was a threshold dose for inducing clinical symptoms and lung function changes in healthy subjects via an inhalation challenge. The threshold dose for inducing changes in blood neutrophils may be less than 0.5 μg LPS. It is not clear how these values might be interpreted in terms of long-term exposure.

## Safety for the farmer and factory worker

Two categories of people may be exposed to endotoxins arising from feed additives, namely farm workers and workers in the premixture factory, i.e. where minerals/vitamins/trace nutrients supplements are prepared. Farmers are routinely exposed to environmental endotoxins, presumably arising from animal faeces. Indeed poultry and pig facilities are among the most hazardous work places in this respect [29]. Little of the endotoxin would originate from feed additives, however. Even if the additive did contain endotoxin, it would already have been mixed with the feed or with a vitamins/minerals pre-mixture. The worker most intimately exposed to the endotoxins in a feed additive would be the worker in the premixture factory.

### Calculation of endotoxin exposure of the premixture factory worker

Two key measurements are required to evaluate the potential respiratory hazard associated with endotoxin in any product, viz*.* the endotoxin activity of the material and the amount of exposure by inhalation.

Several assays are available for measuring endotoxin activity, and it is uncertain which corresponds best to inflammatory potency. Because different LPS molecules have different endotoxin activities, chemical estimation of LPS is not appropriate to assess endotoxin content of additives. Heating of *E. coli* LPS caused inactivation in the *Limulus* amoebocyte lysate (LAL) assay in the same way as inactivation in the TNFα secretion assay [9]. It is recognised that the LAL assay has limitations; however, it is generally accepted for endotoxin measurements in the EU [29, 32]. Different extraction procedures most probably account for a large part of the variation in results [33].

The exposure of workers by inhalation is best measured by personal monitors on workers or by measurements of dust in the work environment. Such data are not always available, so instead it is common to make a conservative estimate of worker exposure from the dusting potential, as measured by the Stauber-Heubach method [34]. The likely exposure time, according to technical guidance of the EFSA FEEDAP Panel [35] for additives added in premixtures, assumes a maximum of 40 periods of exposure per day, each comprising 20 s = 40 × 20 = 800 s per day. With an uncertainty factor of 2, maximum inhalation exposure would occur for 2 × 800 = 1600 s = 0.444 h per day. Again assuming a respiration volume of 1.25 m^3^/h [35], the inhalation volume providing exposure to potentially endotoxin-containing dust would be 0.444 × 1.25 = 0.556 m^3^ per day. If the endotoxin content is *a* IU/g and the dusting potential is *b* g/m^3^, then the endotoxin concentration of the dust would be *a* × *b* IU/m^3^, and exposure to endotoxin in dust would therefore be 0.556 × (*a* × *b*) IU/day (Table 1).

**Table 1:** Estimation of user exposure to endotoxins from feed additives, including consideration of using filter mask FF P2 or FF P3 as preventative measure

Calculation	Identifier	Description	Amount	Source
	a	Endotoxin content IU/g product		
	b	Dusting potential (g/m^3^)		
a × b	c	Endotoxin content in the air (IU/m^3^)		
	d	No of premixture batches made/working day	40	EFSA Guidance on User Safety [35]
	e	Time of exposure (s) per production of one batch	20	EFSA Guidance on User Safety [35]
d × e	f	Total duration of daily exposure/worker (s)	800	
	g	Uncertainty Factor	2	EFSA Guidance on User Safety [35]
f × g	h	Refined total duration of daily exposure/worker (s)	1600	
h/3600	i	Refined total duration of daily exposure (h)	0.444	
	j	Inhaled air (m^3^) per 8-h working day	10	EFSA Guidance on User Safety [35]
j/8 × i	k	Inhaled air during exposure (m^3^)	0.556	
c × k	l	Endotoxin inhaled (IU) during exposure per 8-h working day		
	m	Health based recommended exposure limit of endotoxin (IU/m^3^) per 8-h working day	90	Health Council of the Netherlands [29]
m × j	n	Health based recommended exposure limit of total endotoxin exposure (IU) per 8-h working day	900	
l/10		Endotoxins inhaled (IU) per 8 h working day reduced by filter mask FF P2 (reduction factor 10)		
l/20		Endotoxins inhaled (IU) per 8 h working day reduced by filter mask FF P3 (reduction factor 20)		

### Exposure limits for the factory worker

The Health Council of the Netherlands [29] proposed a health-based recommended exposure limit (HBROEL) of 90 IU/m^3^ (eight-hour time-weighted average) for endotoxins in the workplace. The statutory maximum exposure permitted by the UK Health & Safety Executive [30] is the same. Therefore, the exposure of the factory worker, in the case of feed additives in the premixture unit, should be maintained lower than these nationally respected maxima. Respiration in man may reach 1.25 m^3^/h according to the EFSA FEEDAP panel [35], so inhalation volume over an 8-h working day would be 8 × 1.25 = 10 m^3^. Thus, the maximum permissible total daily exposure by the user, without protection, would be 10 × 90 = 900 IU. The exposure from the endotoxin concentration and dustiness of a product, as calculated above, can then be compared directly with this proposed exposure limit (Table 1).

Rylander [36] concluded that a threshold of 10 ng/m^3^ for an 8-h working day should be applied to prevent lung inflammation in man. Using the assumptions made in Table 1 that the endotoxin activity is 20 IU/ng and the volume of air inhaled in 8 h is 10 m^3^, a value of 10 × 20 × 10 = 2000 IU can be calculated for the total maximum acceptable exposure during an 8-h working day, a value of a similar order of magnitude to the statutory limits adopted above. These values represent a tiny amount of endotoxin, especially when one considers that 1 μL of gut contents can contain >10^3^ IU of soluble endotoxin [20], and factory dust in some industries often exceeds 10^4^ IU/m^3^ [30].

## Safety for farm livestock

### Oral toxicity of LPS

Farm livestock are exposed continuously to endotoxins in their environment [37], including in feed, and to large quantities of LPS present in Gram-negative bacteria in the gastrointestinal tract [25, 38]. Nevertheless, given the potentially toxic effects of endotoxin at small doses, a cautious approach must be taken to possible risks associated with diets containing increased concentrations of endotoxin.

Reports of the consequences of oral ingestion of endotoxin/LPS in farm animals do not seem to be consistent. In contrast to the adverse effects of parenteral administration, for the most part, oral administration of LPS appears to be safe. In a pig study described by the Health Council of the Netherlands [16], high dietary doses of endotoxins did not cause clinical symptoms. Oketani et al. [39] stated that oral administration of LPS is not harmful to animals. Schryvers et al. [40] found no evidence of toxicity when LPS from *Pseudomonas aeruginosa* was added in drinking water for mice (intake 7.2 ml/d), either at a concentration of 20 μg/ml for 40 days or 200 μg/ml for 1 day. Repeated oral administration of high doses of *E. coli* LPS had no demonstrable effect on small intestinal structure and cell proliferation in rats [41]. Taniguchi et al. [42] found that high doses of single oral administration of *Pantoea agglomerans* LPS had no side-effects in rats. Moreover, oral administration of this LPS for 28 days in a repeated-dose study showed no evidence of hepatotoxicity, nephrotoxicity, inflammation, or weight decrease in rats. In their review, Inagawa et al. [17] concluded that these findings demonstrate that oral administration of LPS is safe for animals, although the endotoxin activity of *P. agglomerans* LPS is unclear and the doses used by Taniguchi et al. [42] were not described in a way that enables calculation of daily LPS intake. Furthermore, Inagawa et al. [17] cited literature describing therapeutic effects of *P. agglomerans* LPS in preventing hyperlipidaemia (rabbits), diabetes mellitus (mice and humans), various infectious diseases (mice and shrimps), and ulcerative colitis (mice), as well as causing analgesic effects (mice, rats, and humans). Taniguchi et al. [42] also claimed beneficial effects of oral LPS. In contrast, Cort et al. [43] administered via the feed 40 mg of *Enterobacter agglomerans* LPS to 5 pigs and observed slight to severe signs of endotoxaemia in 3 animals. No effect was seen following intraruminal infusion of up to 20 mg per animal in goats [43].

The other way of assessing the likely hazard posed by an endotoxin-containing feed additive is – does the additive increase significantly the amount of LPS that may be ingested in the normal course of events in a typical farm? Animal feed may be contaminated with endotoxin on a regular basis (Table 2). Cort et al. [43] reported concentrations of 12.4 and 12.9 ng endotoxin/mg, assayed by the LAL assay, in pig feed and cited other analyses where values up to 60 ng/mg were found. Details of the methods of extraction were not provided. The same authors measured 0.05 ng/mg in hay and 10 ng/mg in pelleted feed for goats. Corn silage contained 1 IU endotoxin/mg in samples from the centre of the silo, but concentrations 200× higher were found in samples taken from the surface [44]. Two feed samples from a duck-fattening farm contained 50 and 93 IU/mg [45]. In a study of feedstuffs for horses by Wolf et al. [46], 47 % of oats samples and 73 % of straw samples contained >50 ng endotoxin/mg, or >1000 IU endotoxin/mg. Similar contamination of horse feed materials was observed by Kamphues et al. [47]. Ratzinger [48] reported a range of 7.5–259 (mean 64.7) ng/mg in pig feed samples from 16 different farms. Liebers et al. [49] used a value of 10 IU endotoxin/ng LPS. Thus, the endotoxin concentration in the horse feeds would be 75–2590 IU/mg.

**Table 2:** Endotoxin contamination of feed materials

Feed material	Average/max concentration (units quoted)	Average/max concentration (IU/mg, calculated)^a^	Reference
Pig feed	13/60 mg/kg	260/1200	Cort et al. [43]
Hay	0.05 mg/kg	1	Cort et al. [43]
Pelleted goat feed	10 mg/kg	200	Cort et al. [43]
Corn silage	1/200 IU/mg	1/200	Dutkiewicz et al. [44]
Duck feed	50, 93 IU/mg	50, 93	Scharf [45]
Horse feed oats	>50 ng/mg	>1000	Wolf et al. [46]
Pig feed	7.5-259, mean 64.7, ng/mg	150-5,180, mean 1294	Ratzinger [48]

^a^Using an activity of 20 IU/ng pure endotoxin, based on values of 12–25 IU/ng estimated by Luchi and Morrison [7] and 10 IU/ng by Liebers et al. [49]

It can be concluded, therefore, that normal feedstuffs may be contaminated with varying concentrations of endotoxins, with values of 1000 IU/mg feed not being unusual, so it can be concluded that oral ingestion of endotoxin in small quantities would not be harmful, and would occur normally as a consequence of the consumption of feedstuffs. Providing the endotoxin concentration in additives produced by fermentation does not exceed these concentrations, no additional risk to the target animal would be anticipated. Furthermore, because ruminants already harbour large amounts of endotoxin in ruminal digesta, they appear likely to be more resilient to endotoxin contamination of the feed. The exception would be animals in which gastrointestinal disturbance has compromised the barrier function of gut tissues. In these animals, excluding any endotoxin-containing feed ingredients, if possible, would be recommended.

### Inhalation toxicity of endotoxin in the animal

A dusty endotoxin-contaminated feed could presumably lead to similar problems in target animals to those described for the human user. The sensitivity of factory workers to inhalation of endotoxin from the feed additive prompts the consideration that the animal consuming a diet containing an endotoxin-contaminated additive might similarly be exposed to hazard by inhalation. Only one report was found that indicated respiratory problems associated with farm livestock consuming endotoxin-contaminated feed. Horses consuming feed contaminated by >50 ng endotoxin/mg feed suffered reduced feed intake, increased incidence of respiratory diseases, and elevated body temperature, sudden death, allergic skin reactions and reduced mobile capacity [47]. LPS induced lung injury in rats, although the dose was very high (100 mg/m^3^ over 6 h; [50]). In mice, repeated low-dose LPS inhalation resulted in airway hyperresponsiveness, associated with a failure to resolve the proinflammatory response, an inverted macrophage to dendritic cell ratio, and a significant rise in the inflammatory dendritic cell population [51]. A No Observed Adverse Effect Concentration (NOAEC) was not identified in any of these studies. The paucity of data indicate that there is a need for research to provide answers as to the effect on animal welfare of the inhalation of endotoxins by farm animals, both from feed and from feed additives.

In the absence of information or guidance on how inhalation exposure for animals might be calculated, a similar approach to ingestion exposure would be prudent, namely to assume that feed additives with an endotoxin concentration comparable to that found in feed would not be expected to result in any increased hazard. Nonetheless, it seems advisable to monitor animals receiving feed additives produced by *E. coli* for symptoms of increased respiratory stress.

## Safety for the consumer

In healthy individuals, LPS does not cross the intestinal barrier easily [52–55] and LPS is metabolised in animal tissues, particularly the liver [56], and in the lung [25, 29]. Endotoxins that reach the respiratory tract are rendered harmless by macrophages and polymorphonuclear leukocytes [25, 29]. Thus, endotoxin would not be expected to accumulate in edible tissues. An endotoxin-contaminated feed ingredient would therefore not pose a risk to the consumer of animal products.

## Conclusions

- In order that the risk to factory workers caused by endotoxin contamination of additives produced by fermentation using *E. coli* or other Gram-negative bacteria can be assessed, data on dusting potential (expressed in g/m^3^, preferably by the Stauber-Heubach method) should be provided. Dusting potential and endotoxin activity (LAL assay) can be used to assess the risk to workers by inhalation.
- If the results of this conservative estimate of worker exposure indicate that inhalation exposure would be less than the health-based occupation exposure limit, it can be concluded that there will be no health risk for workers. If, however, the results estimate that exposure may be in excess of the limit, it must be assumed that workers are at risk, unless more refined measurements of exposure can be produced to demonstrate that inhalation exposure of workers is below the limit.
- To ensure animal safety by oral administration or by inhalation of dust, when an additive produced by Gram-negative bacteria is added to the feed at the proposed use level, the additional endotoxin concentration of the feed resulting from the additive should not exceed 1000 IU/mg.

## Abbreviations

EFSA: European Food Safety Authority
FEEDAP: EFSA Panel on Additives and Products or Substances used in Animal Feed
HBROEL: health-based recommended exposure limit
IU: international endotoxin units
LAL: *Limulus* amoebocyte lysate
LPS: lipopolysaccharide
